# A Cure for Dandruff

**Published:** 1890-05

**Authors:** A. J. Harrison

**Affiliations:** Bristol, England


A CURE FOR DANDRUFF.—Dr. A. J. Harrison, Bristol, England:

R. Caustic potash, 8 grains.
Phenic acid, 24 grains.
Lanolin.
Cocoanut oil, aa ℥ iv. M.

Sig. This preparation should be rubbed into the scalp morning and evening. Complete cure is usually effected in one to three months.

---

It is stated that one grain of pilocarpine in a half ounce of vaseline applied to the scalp will prevent baldness.—*Gaillard's Medical Journal.*

---

Quinolineparamethenylbenzenyparacarboxylic acid is a new medicinal agent, made by melting quinolinesparamethenylinamidoxine with pthalic anhydride. It is *not* recommended for lockjaw.—*Medical World.*

